# *Lactobacillus rhamnosus* GG Protects the Epithelial Barrier of Wistar Rats from the Pepsin-Trypsin-Digested Gliadin (PTG)-Induced Enteropathy

**DOI:** 10.3390/nu10111698

**Published:** 2018-11-07

**Authors:** Antonella Orlando, Michele Linsalata, Giusy Bianco, Maria Notarnicola, Benedetta D’Attoma, Maria Principia Scavo, Angela Tafaro, Francesco Russo

**Affiliations:** 1 Laboratory of Nutritional Pathophysiology, National Institute of Gastroenterology “S. de Bellis” Research Hospital, 70013 Castellana Grotte (Ba), Italy; antonella.orlando@irccsdebellis.it (A.O.); michele.linsalata@irccsdebellis.it (M.L.); benedetta.dattoma@irccsdebellis.it (B.D.); 2 Animal Facility, National Institute of Gastroenterology “S. de Bellis” Research Hospital, 70013 Castellana Grotte (Ba), Italy; giusy.bianco@icloud.com (G.B.); angela.tafaro@irccsdebellis.it (A.T.); 3 Laboratory of Nutritional Biochemistry, National Institute of Gastroenterology “S. de Bellis” Research Hospital, 70013 Castellana Grotte (Ba), Italy; maria.notarnicola@irccsdebellis.it; 4 National Institute of Gastroenterology “S. de Bellis” Research Hospital, 70013 Castellana Grotte (Ba), Italy; maria.scavo@irccsdebellis.it

**Keywords:** adherens junctions, celiac disease, gliadin, intestinal permeability, polyamines, probiotics, tight junctions

## Abstract

Celiac disease (CD) is a chronic immune-mediated disorder, characterized by enhanced paracellular permeability across the intestinal epithelium. The complex system of intercellular junctions, including tight junctions (TJs) and adherens junctions (AJs), seals together the epithelial cells to form a continuous layer. The improvements in barrier integrity have been related to modifications in intercellular junction protein expression. Polyamines (spermidine, spermine, and putrescine) actively participate in the modulation of the AJ expression. Both in vitro and in vivo studies have demonstrated that also probiotics can promote the integrity and the function of the intestinal barrier. On these bases, the present work investigated the protective effects exerted by *Lactobacillus rhamnosus* GG (L.GG) against the pepsin-trypsin-digested gliadin (PTG)-induced enteropathy in jejunal tissue samples of Wistar rats. In particular, the probiotic effects have been evaluated on the intestinal mucosal architecture, polyamine metabolism and intercellular junction protein expression (ZO-1, Occludin, Claudin-1, β-catenin and E-cadherin). The results from this study indicate that L.GG protects the intestinal mucosa of rats from PTG-induced damage, by preventing the reduction of the expression of the intercellular junction proteins. Consequently, a role for L.GG in the therapeutic management of the gluten-related disorders in humans could be hypothesized.

## 1. Introduction

The ingestion of gluten can cause the onset of celiac disease (CD) in genetically predisposed subjects [1]. This disease is a chronic immune-mediated disorder, characterized by enhanced paracellular permeability across the intestinal epithelium [2]. The gliadin fraction of wheat gluten is responsible for the development of the CD typical intestinal damage and the gliadin deamidation by tissue transglutaminase has been suggested to represent the first step into the sequence of autoimmune reactions leading to alterations of the integrity of the intestinal barrier and finally to the mucosal destruction [3].

The main role of the intestinal barrier is to divide the internal environment from the luminal content [4], and the complex system of intercellular junctions, including tight junctions (TJs), adherens junctions (AJs), desmosomes, and gap junctions [5], seals together the epithelial cells to form a continuous layer. In health conditions, the intestinal barrier is almost impermeable to macromolecules. TJs are highly dynamic structures located near the apical surface of enterocytes, with several transmembrane proteins contributing to their composition. Occludin, claudins, junction adhesion molecules, and scaffold proteins—zonula occludens (ZO-1, ZO-2, and ZO-3) all represent active parts of these dynamic structures. Beneath the TJs are positioned the AJs with their rich content in cadherin that mediate strong cell-to-cell adhesion. In turn, dense peri-junctional rings, formed of actin and myosin, support TJs and AJs in forming the apical junctional complex and regulating the epithelial paracellular permeability [6].

Among the several substances involved in the control of the intestinal permeability, polyamines play a crucial role. Polyamines (including spermidine, spermine, and their precursor putrescine) are organic cations, found in all the eukaryotic cells, involved in a plethora of biological functions; their regulation at the cellular level has been recognized to be essential for multiple signaling pathways driving cell functions such as cell motility, proliferation, and apoptosis [7].

Polyamines also participate in the regulation of intestinal epithelial barrier function by regulating the intercellular junctions levels. Their depletion, obtained by inhibiting their limiting enzyme ornithine decarboxylase (ODC), can cause, together with other mechanisms, the reduction in the levels of some AJ proteins such as E-cadherin, ß-catenin, and α-catenin [8]. The modulation of the expression of various intercellular junction proteins occurs through distinct cellular signaling pathways, although not completely elucidated.

Both in vitro and in vivo studies have demonstrated that also probiotics can promote the integrity and the function of intestinal barrier by contrasting the action of cytokines, toxins, and pathogens in the disruption of TJ complexes and by contemporary improving the TJ stability [9,10]. *L. plantarum* has been reported to confer protection against chemically induced rupture of the epithelial barrier in vitro, and to regulate in vivo TJ proteins of the human epithelium [11]. *L. plantarum* administered to healthy volunteers caused a significant increase of ZO-1 and occludin proteins in the small intestine. Along with *L. plantarum*, other Lactobacillus strains (e.g., *L. rhamnosus* GG, *L. salivarius* strains UCC118 and CCUG38008, *L. casei* strain DN—114 001, and *L. casei* Shirota) have been demonstrated in different in vitro and in vivo models to exert protective effects on the intestinal barrier [12,13,14,15].

The initial steps that allow gliadin to cross the intestinal barrier to be recognized by the intestinal immune system are still under investigation. Some of the effects reported in the literature have been ascribed not to gliadin itself but, instead, to peptic tryptic digests [16]. Thus, different in vitro studies have been performed using gliadin peptides, such as pepsin-trypsin-digested gliadin (PTG). In this context, previous works by our group demonstrated that the administration of wheat gliadin or PTG to Caco-2 cell line caused significant alterations of the paracellular permeability and the L.GG strain had the potential to restore the cell barrier function [17,18].

Several attempts have been made to set up animal models that could reproduce all the aspects linked to CD, including the pathogenesis, the immune response, the mucosal lesions, and the symptoms [19,20,21], although without obtaining unequivocal results. Some of the proposed models were based on the intragastric administration of gliadin to inbred rats after weaning [20] or to immunocompetent mice at ten days of age; however, they failed in reproducing damages of the intestinal mucosa resembling those in CD [22]. In another experimental design, the immune response to gliadin, involving both the adaptive and innate immune system, was developed in transgenic mice sensitized with gluten. These animals also developed changes in gut neuromuscular and epithelial secretory function, but without showing a real gluten-dependent enteropathy [23,24]. On the contrary, a procedure based on the recurring administration of gliadin orally to rats previously sensitized with interferon gamma (IFN-γ) immediately after birth proved to mimic in vivo the CD lesions successfully [25]. This procedure can induce a CD4+ T cell-mediated enteropathy, defined as hyperplasic-infiltrative (type II), resembling that described in CD patients.

The present study was aimed at evaluating the potential protective effects exerted by L.GG treatment against the PTG-induced enteropathy in jejunal tissue samples of Wistar rats. On these bases, the animals were treated by applying the above-cited model [25], and the probiotic effects were evaluated on the intestinal mucosal architecture, polyamine metabolism and intercellular junction protein expression (namely ZO-1, Occludin, Claudin-1, β-catenin and E-cadherin). 

## 2. Materials and Methods 

### 2.1. Gliadin Digest

To obtain PTG, commercially available gliadin was digested as previously described by Drago et al. [26]. Briefly, 50 g wheat gliadin (Sigma-Aldrich, Milan, Italy) was dissolved in 500 mL 0.2 N HCl for 2 h at 37 °C with 1 g pepsin (Sigma-Aldrich, Milan, Italy). The resultant peptic digest was further digested by addition of 1 g trypsin (Sigma-Aldrich, Milan, Italy) after pH adjusted to 7.4 using NaOH 2 M. The solution was stirred vigorously at 37 °C for 4 h, and then boiled (100 °C) for 30 min, freeze-dried, lyophilized, and stored at −20 °C until used.

### 2.2. Animals and Experimental Design

In vivo experiments were carried out using new-born Wistar rats. The procedures related to animal use have been approved by the Italian Ministry of Health (approval date: 15 December 2016; n. 1178/2016-PR) and conducted in adherence with the International Guidelines for the use of laboratory animals.

Every litter of at least ten puppies represented a different treatment group. In detail: “Ctrl” (without treatment); “PTG” (sensitized with 1000 U IFN-γ administered intraperitoneally after birth and treated with PTG 50 µg/day for 10 days); “L.GG” (treated with L.GG 1 × 10^9^ CFU for 10 days); “Co-administered” (sensitized with 1000 U IFN-γ administered intraperitoneally after birth and co-administered with PTG and L.GG for 10 days); “Pre-treated” (sensitized with 1000 U IFN-γ administered intraperitoneally after birth, pre-treated with PTG for 10 days, and then administered with L.GG for further 10 days).

As concerns PTG treatment, new-born rats received 50 µg PTG/day in a single dose for 10 days, and, finally, a provocative dose of PTG 100 µg two hours before sacrifice.

The probiotic *Lactobacillus rhamnosus* GG (ATCC 53103) (Dicoflor, Dicofarm, Rome, Italy) was administered at a concentration of 10^9^ CFU/day in a single dose for 10 days.

Changes in body weight were monitored every two days. After treatments, the puppies were sacrificed by anesthetic overdose, and the jejunal tissue samples were immediately removed and stored at −80 °C until assayed.

### 2.3. Histologic and Morphometric Evaluation

Jejunal tissue sections were fixed in formaldehyde 10% and then, to evaluate the grade of the damage, hematoxylin-eosin stained sections were examined in a blinded fashion from the operator. The damage was defined as the occurrence of disorganized glandular and villi architecture, similarly to the technique commonly applied in human intestinal biopsies. The samples were analysed with Nikon Eclipse Ti2 equipped with DCiN-12V digital camera. All images were analysed with Nis Elements software (Nikon Instruments Inc., Melville, NY, USA). The analysis considered the length and width of the villi, because characterized the histologic modification during the enteropathy evolution. The eventual presence of inflammatory cells that indicate the disorder of the enteric mucosa has also been considered.

### 2.4. Polyamine Analysis

For the evaluation of polyamine levels, after rats were subjected to the above described different treatments, each jejunal tissue sample was homogenized in 700 µL of 0.9% sodium chloride mixed with 10 µL (200 nmol/mL) of the internal standard 1,10-diaminodecane (1,10-DAD). An aliquot of the homogenate was used to measure the total protein content. Then, to precipitate proteins, 50 µL of perchloride acid (PCA) 3 M were added to the homogenate. After 30 min of incubation in ice, the homogenate was centrifuged for 15 min at 7000× *g*. The supernatant was filtered (Millex-HV13 pore size 0.45 µm, Millipore, Bedford, MA, USA) and lyophilized. The residue was dissolved in 300 µL of HCl (0.1 N). Dansylation and the extraction of dansyl-polyamine derivatives were performed as previously described [27]. After extraction, aliquots of 200 µL were injected into a high-performance liquid chromatography system (UltiMate 3000, Dionex Corp., Sunnyvale, CA, USA) equipped with a reverse-phase column (Sunfire C18, 4.6 × 100 mm, 3.5 µm particle size, Waters, Milford, MA, USA). Polyamines were eluted with a linear gradient ranging from acetonitrile-water (50:50, *v*:*v*) to acetonitrile (100%) for 30 min. The flow was 0.5–1.0 mL/min from 0 to 12 min and then set at a constant rate (1.0 mL/min) until the 30th min. The fluorescent intensity was monitored by a fluorescence detector (UltiMate 3000 RS, Dionex Corp., Sunnyvale, CA, USA) with excitation at 320 nm and emission at 512 nm. Polyamine levels were expressed as concentration values in nmol/mg of protein.

### 2.5. ODC Activity

The effects of the above described different treatments on ODC activity in each jejunal tissue sample was investigated by a radiometric technique that estimated the amount of ^14^CO_2_ liberated from DL-[1-^14^C]-ornithine (specific activity, 56.0 mCi/mmol; Hartmann Analytic, Braunschweig, Germany) [28]. The jejunal tissue sample was homogenized in 0.6 mL ice-cold Tris-HCl (15 mM, pH 7.5) containing 2.5 mM dithiothreitol, 40 µM pyridoxal-5-phosphate, and 100 µM ethylene diamine tetra acetate and then centrifuged at 30,000× *g* for 30 min at 4 °C. An aliquot of supernatant (200 µL) was added to a glass test tube containing 0.05 µCi DL-[1-^14^C]-ornithine and 39 nmol DL-ornithine. After incubation for 60 min at 37 °C, the reaction was stopped by adding trichloroacetic acid (TCA) to a final concentration of 50%. ^14^CO_2_ liberated from DL-[1-^14^C]-ornithine was trapped on filter paper pre-treated with 40 µL NaOH (2 N), which was suspended in a center well above the reaction mixture. Radioactivity on the filter papers was determined by a liquid scintillation counter (model 1219 Rackbeta; Hidex 300SL, Wien, Austria). ODC activity was expressed as pmolCO_2_/h/mg of protein. Enzymatic activity was found to be linear within the range of 50–600 µg of protein (r^2^ = 0.99). The intra-assay and inter-assay variation coefficients (CV%) were 4% and 7%, respectively.

### 2.6. Real-Time PCR

The effects of the above described different treatments on ZO-1, Occludin, Claudin-1, β-catenin, E-cadherin mRNA in each jejunal tissue sample were evaluated using the quantitative PCR (qPCR) method with SYBR1 green dye. 

The jejunal tissue samples were re-suspended in 0.3 mL of pure distilled water and used for RNA extraction. Total cell RNA was extracted using Tri-Reagent (Mol. Res. Center Inc., Cincinnati, OH, USA), following the manufacture’s instruction. About 2 μg total cell RNA, extracted from both the control and treated tissue samples, was used for cDNA synthesis. Reverse transcription (RT) was carried out in 20 μL of the final volume at 42 °C for 30 min, using the iScript Advanced cDNA Synthesis Kit (Bio-Rad, Milan, Italy). Real-time PCRs were performed in 25 µL of a final volume containing 2 µL of cDNA, master mix with SYBR Green (iQ SYBR Green Supermix, Bio-Rad, Milan, Italy) and sense and antisense primers for the ZO-1, Occludin, Claudin-1, E-cadherin, ß-catenin, and the ß-actin gene. The ß-actin gene was used as an internal control and was chosen as a reference gene because it is a housekeeping gene.

Real-time PCRs were carried out in a CFX96 Real-Time PCR Detection System (Bio-Rad, Milan, Italy) using the following protocol: 45 cycles at 95 °C for 3 min, 95 °C for 10 s, 55 °C for 30 s followed by a melting curve step at 65–95 °C with a heating rate of 0.5 °C per cycle for 80 cycles. Relative quantification was done using the ∆∆Ct method.

### 2.7. Western Blotting

Protein extracts were obtained treating each jejunal tissue sample from control and treated rats with total lysis buffer (Pierce Ripa buffer, Thermo Scientific, Rockford, IL, USA) supplemented with protease and phosphatase inhibitors (Thermo Scientific, Rockford, IL, USA).

After homogenization and centrifugation at 14,000 rpm for 15 min at 4 °C, protein concentration was measured by a standard Bradford assay (Bio-Rad, Milan, Italy). Aliquots of 50 µg of total protein extracts from each sample were denaturated in 5× Laemmli sample buffer and loaded into 4–12% pre-cast polyacrylamide gels (Bio-Rad, Milan, Italy) for western blot analysis. ODC (N-15), ZO-1 (C-19), Occludin (H-279), Claudin-1 (C-18), β-catenin (H-102), p-β-catenin (Ser 33), E-cadherin (H-108) and β-actin (Santa Cruz Biotechnology, Santa Cruz, CA, USA) were used as primary antibodies. After overnight incubation, the membranes were further incubated with a horseradish peroxidase-conjugated goat secondary antibody (Bio-Rad, Milan, Italy). The proteins were detected by chemiluminescence (ECL, Thermo Scientific, Rockford, IL, USA) and the densitometric analysis of each protein-related signal was obtained using the Molecular Imager ChemidocTM (Bio-Rad, Milan, Italy) and normalized against β-actin expression.

### 2.8. Statistical Analysis

Due to the non-normal distribution of the data, non-parametric tests were performed. Data were analyzed by Kruskal-Wallis analysis of variance and Dunn’s Multiple Comparison Test. All data are expressed as mean and SEM. Differences were considered significant at *p* < 0.05. A specific software package (SigmaStat for Windows version 3.00 SPSS Inc. San Jose, CA, USA) was used.

## 3. Results

### 3.1. Morphometric Analysis

**Figure 1:**
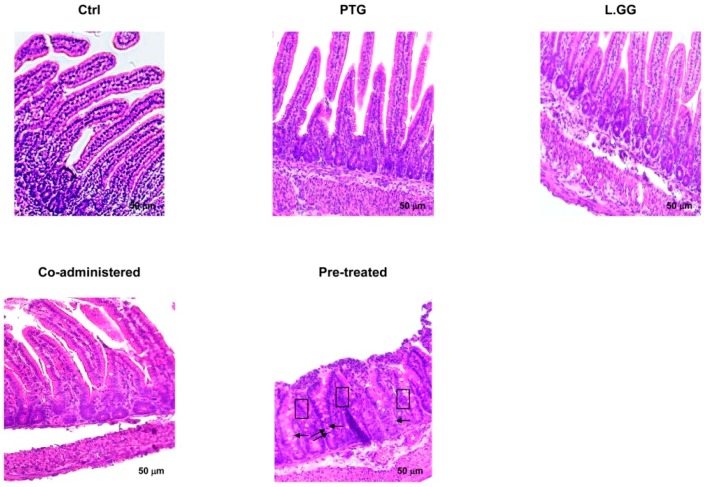
Histology of jejunal tissue sections from control and treated rats, stained with hematoxylin-eosin. Ctrl (without treatment); PTG (sensitized with 1000 U IFN-γ and treated with PTG 50 µg/day for ten days); L.GG (treated with L.GG 1 × 10^9^ CFU for ten days); Co-administered (sensitized with 1000 U IFN-γ and co-administered with PTG and L.GG for ten days); Pre-treated (sensitized with 1000 U IFN-γ, pre-treated with PTG for ten days, and then administered with L.GG for further ten days). Scale bar, 50 µm.

Figure 1 reports the histological study of jejunal tissue sections from control and treated rats. 

The animals sensitized with IFN-γ and treated with PTG for ten days showed a significant (*p* < 0.01) 44.3% reduction in the length of villi, compared to the control mucosa (Table 1). On the contrary, when the rats were treated with L.GG for ten days, no significant difference in the morphology of villi resulted (Table 1). The co-administration of PTG and L.GG for ten days induced a partial restoration of the mucosa with a significant (*p* < 0.05) 53.5% increase in the length of villi compared to PTG treated rats (Table 1). Finally, in rats sensitized with IFN-γ, pre-treated with PTG for ten days and then administered with L.GG for further ten days, an injured mucosa with some inflammatory cells between the villi and on the surface of the mucosa was observed (highlighted by black arrows in Figure 1). A significant (*p* < 0.01) 38.6% reduction in the length of villi was found in this group of animals compared to control rats (Table 1). No increase in thickness was observed, although some areas of the mucosa showed a cellular double layer (highlighted by the black box in Figure 1).

### 3.2. Polyamine Profile

The polyamine profile, expressed as nmol/mg of protein, in jejunal tissue samples from controls and treated rats is shown in Table 2. The administration of PTG to rats sensitized with IFN-γ, as well as the exposure to L.GG had no significant effect on both the single and total polyamine levels. When L.GG was co-administered with PTG to rats sensitized with IFN-γ, a significant (*p* < 0.05) decrease in the spermidine (−28.2%) and total polyamine content (−16.0%) occurred, compared to PTG treated rats. The addition of L.GG for further ten days to rats sensitized with IFN-γ and pre-treated with PTG for ten days, caused a significant (*p* < 0.05) reduction in the total polyamine levels (−16.6%) in comparison with PTG treated rats. 

### 3.3. ODC Activity

**Figure 2:**
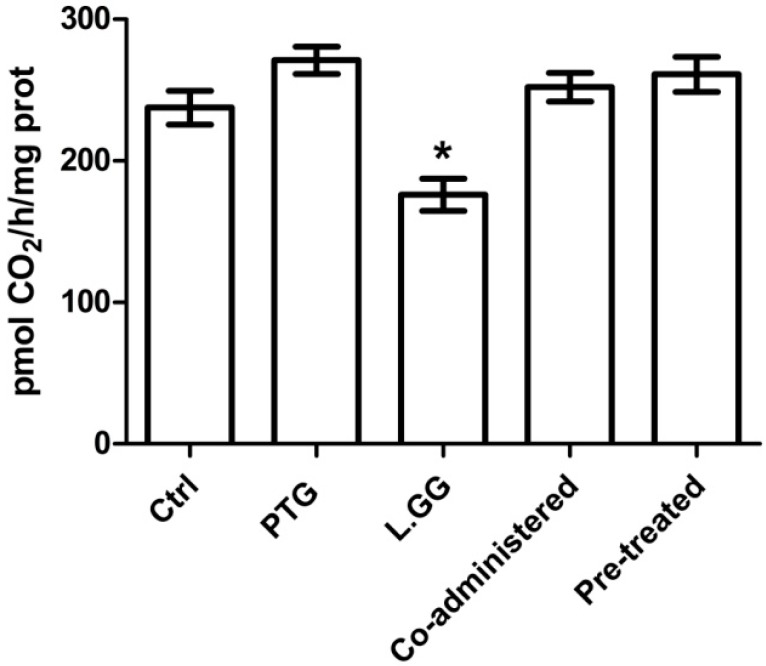
Ornithine Decarboxylase (ODC) activity in jejunal tissue samples from control and treated rats. Ctrl (without treatment); PTG (sensitized with 1000 U IFN-γ and treated with PTG 50 µg/day for ten days); L.GG (treated with L.GG 1 × 10^9^ CFU for ten days); Co-administered (sensitized with 1000 U IFN-γ and co-administered with PTG and L.GG for ten days); Pre-treated (sensitized with 1000 U IFN-γ, pre-treated with PTG for ten days, and then administered with L.GG for further ten days). All data represent the results of three different experiments (mean ± SEM). ODC activity was expressed as pmol CO_2_/h/mg of protein. Data were analysed by Kruskal-Wallis analysis of variance and Dunn’s Multiple Comparison Test. (* *p* < 0.05 compared to control rats).

Figure 2 shows the ODC activity, expressed as pmol CO_2_/h/mg of protein, in jejunal tissue samples from all the rats. The administration of PTG to rats sensitized with IFN-γ had no significant effect on the enzymatic activity. By opposite, L.GG treatment caused a significant (*p* < 0.05) decrease (−25.9%) in the ODC activity in comparison with control rats (237.5 ± 11.9 vs. 176.0 ± 11.5). The co-administration of PTG and L.GG to rats sensitized with IFN-γ, as well as the addition of L.GG for further ten days to rats sensitized with IFN-γ and pre-treated with PTG for ten days caused no significant effect.

### 3.4. ODC Expression

**Figure 3:**
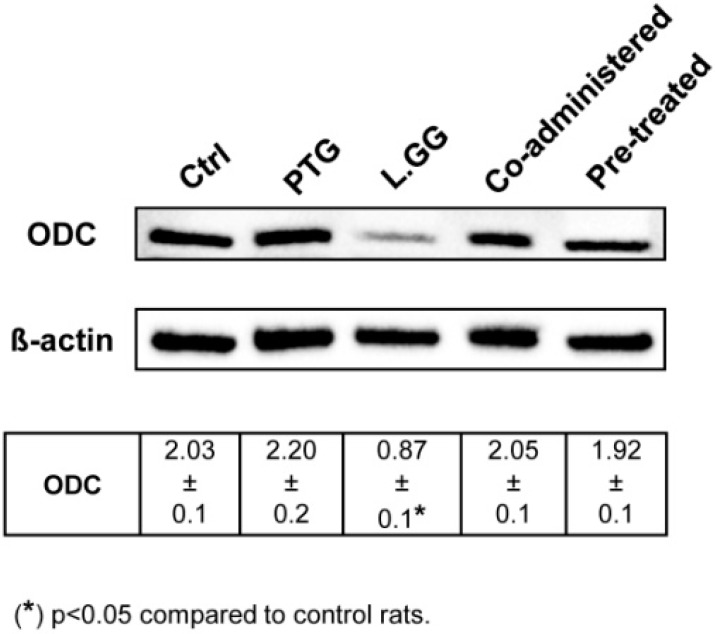
Western Blot Analysis of ODC in jejunal tissue samples from control and treated rats. Ctrl (without treatment); PTG (sensitized with 1000 U IFN-γ and treated with PTG 50 µg/day for ten days); L.GG (treated with L.GG 1 × 10^9^ CFU for ten days); Co-administered (sensitized with 1000 U IFN-γ and co-administered with PTG and L.GG for ten days); Pre-treated (sensitized with 1000 U IFN-γ, pre-treated with PTG for ten days, and then administered with L.GG for further ten days). Immunoreactive bands were quantified using Quantity One Program. The table shows quantification of the intensity of bands, calibrated to the intensity of the β-actin band. All data represent the results of three different experiments (mean ± SEM). Data were analyzed by Kruskal-Wallis analysis of variance and Dunn’s Multiple Comparison Test.

ODC expression, reported as intensity of bands, in jejunal tissue samples from control and treated rats was evaluated by Western Blot analysis (Figure 3). PTG treatment of rats sensitized with IFN-γ had no significant effect on the ODC protein levels. By opposite, the administration of L.GG to rats caused a significant (*p* < 0.05) ODC reduction (−57.1%) in comparison with untreated rats. The co-administration of PTG and L.GG to rats sensitized with IFN-γ, as well as the pre-treatment with PTG for ten days, followed by the exposure to L.GG for further ten days had no significant effect on the ODC protein content.

### 3.5. Intercellular Junction Protein Expression

**Figure 4:**
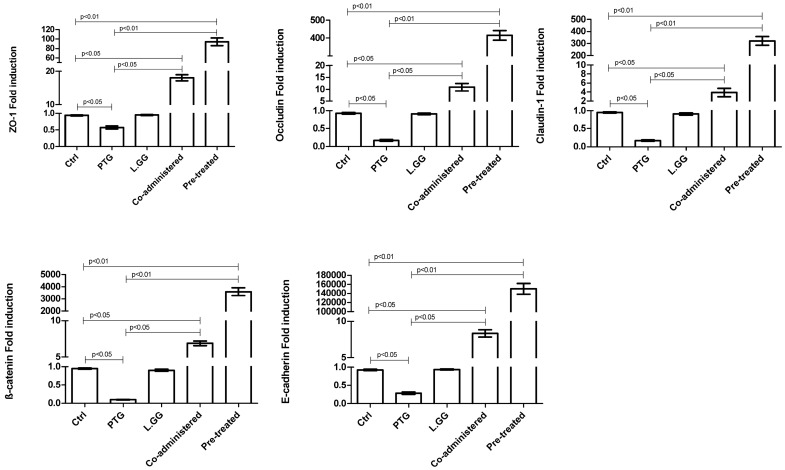
Transcription analysis of ZO-1, Occludin, Claudin-1, β-catenin and E-cadherin genes by qPCR in jejunal tissue samples from control and treated rats. Ctrl (without treatment); PTG (sensitized with 1000 U IFN-γ and treated with PTG 50 µg/day for ten days); L.GG (treated with L.GG 1 × 10^9^ CFU for ten days); Co-administered (sensitized with 1000 U IFN-γ and co-administered with PTG and L.GG for ten days); Pre-treated (sensitized with 1000 U IFN-γ, pre-treated with PTG for ten days, and then administered with L.GG for further ten days). All data represent the results of three different experiments (mean ± SEM). Data were expressed as Fold induction. Data were analysed by Kruskal-Wallis analysis of variance and Dunn’s Multiple Comparison Test.

**Figure 5:**
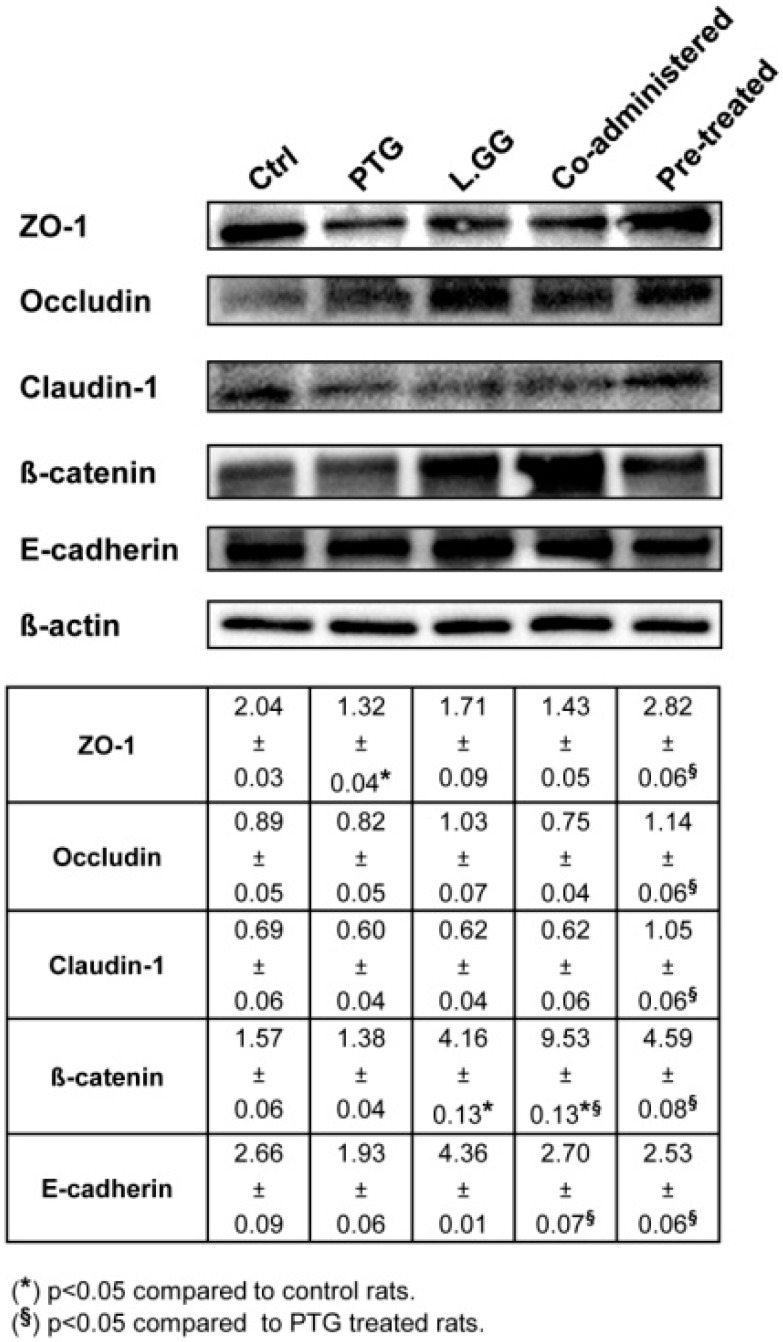
Western Blot Analysis of ZO-1, Occludin, Claudin-1, β-catenin and E-cadherin in jejunal tissue samples from control and treated rats. Ctrl (without treatment); PTG (sensitized with 1000 U IFN-γ and treated with PTG 50 µg/day for ten days); L.GG (treated with L.GG 1 × 10^9^ CFU for ten days); Co-administered (sensitized with 1000 U IFN-γ and co-administered with PTG and L.GG for ten days); Pre-treated (sensitized with 1000 U IFN-γ, pre-treated with PTG for ten days, and then administered with L.GG for further ten days). Immunoreactive bands were quantified using Quantity One Program. The table shows quantification of the intensity of bands, calibrated to the intensity of the β-actin band. All data represent the results of three different experiments (mean ± SEM). Data were analysed by Kruskal-Wallis analysis of variance and Dunn’s Multiple Comparison Test.

ZO-1, Occludin, Claudin-1, ß-catenin and E-cadherin mRNAs and protein levels (expressed as fold induction and intensity of the bands, respectively) in jejunal tissue samples from control and treated rats were quantified by qPCR (Figure 4) and Western Blot analysis (Figure 5), respectively. The exposure to PTG of rats sensitized with IFN-γ led to a significant (*p* < 0.05) reduction in the expression of all the tested genes (Figure 4). In particular, ZO-1, Occludin, Claudin-1, ß-catenin and E-cadherin caused a fold induction by 0.57, 0.17, 0.17, 0.10 and 0.28, respectively, in comparison with control rats. The administration of L.GG to rats did not cause any significant effect. The co-administration of PTG and L.GG to rats sensitized with IFN-γ caused a significant (*p* < 0.05) fold induction of ZO-1 (18.09), Occludin (10.87), Claudin-1 (3.88), ß-catenin (6.91) and E-cadherin (8.33) genes, in comparison with both control rats and PTG treated ones. The addition of L.GG for ten days to rats sensitized with IFN-γ and pre-treated with PTG for further ten days, induced dramatically and significantly (*p* < 0.01) ZO-1, Occludin, Claudin-1, ß-catenin and E-cadherin gene expression, with a fold induction by 93.76, 415.10, 320.20, 3.59 × 10^3^, and 150.19 × 10^3^, respectively, compared to both control rats and PTG treated ones (Figure 4). 

As concerns Western Blot analysis, PTG administration to rats sensitized with IFN-γ led to a significant (*p* < 0.05) reduction only in the ZO-1 (−35.3%) protein levels compared to control rats (Figure 5). The treatment with L.GG caused a significant (*p* < 0.05) increase in the ß-catenin (+165.0%) protein levels compared to untreated rats. When PTG and L.GG were co-administered to rats sensitized with IFN-γ, a significant (*p* < 0.05) enhancement in the ß-catenin (+590.6%) and E-cadherin (+39.9%) proteins compared to PTG treated rats was observed. The increase of ß-catenin protein (+507.0%) resulted significant (*p* < 0.05) also in comparison with untreated rats. Lastly, the addition of L.GG for ten days to rats sensitized with IFN-γ and pre-treated with PTG for further ten days, led to a significant (*p* < 0.05) increase in all the tested intercellular junction protein levels. In detail, ZO-1 increased by 113.6%, Occludin by 39.0%, Claudin-1 by 75.0%, ß-catenin by 232.6%, and E-cadherin by 31.1% compared to PTG treated rats.

## 4. Discussion

The ingestion of gliadins in susceptible individuals can cause the CD onset. The disease is characterized by the destruction of the villus structure in the duodenal mucosa induced by gliadins that can trigger TH-1 dependent inflammatory reactions after they have passed the intestinal epithelial border [1].

The paracellular space is sealed by TJs which regulate the flow of water ions and small molecules through different types of claudins and other proteins in the junctional complex. The assembly of the TJs is facilitated by the formation of the AJs in which the classical cadherins form a basic complex with the catenins [29].

In the absence of inflammation or epithelial disruption, the functional state of the TJs determines paracellular permeability. TJs are highly dynamic, opening and closing in response to the cytoskeletal reorganization that occurs upon exposure to external antigens, as it happens for gliadins in CD [30]. Gliadins induce a loss of barrier function and stimulate innate and adaptive immune responses, followed by intestinal damage. These proteins also disrupt TJ integrity by altering actin and ZO-1 distribution in intestinal epithelial cells [31]. Besides gliadins can also cause alterations of ZO-1 phosphorylation and expression [32]. 

In the present study, we aimed at evaluating further in an experimental model of PTG-induced enteropathy, the protective effect of L.GG on the intestinal mucosal architecture, polyamine metabolism and intercellular junction protein expression in jejunal tissue samples of Wistar rats. The model we chose was that of Laparra et al. [25] since, after several efforts, these authors set up a reliable model to mimic a CD histological pattern. In their study, laboratory animals sensitized with IFN-γ and exposed to gliadin, suffered from mucosal damage and immunological changes resembling those observed in the human CD. The sensitization of animals with IFN-γ appeared to be necessary for the full establishment of a jejunal mucosal reaction and the instauration of the enteropathy [33]. This animal model reproduced a CD4+ T cell-mediated enteropathy, defined as hyperplasic-infiltrative (type II), similar to that described in CD patients. It has been deemed appropriate to initially explore pathogenic mechanisms, as well as potential pharmaceutical or nutritional interventions. Nevertheless, further refinement of this model of CD is at the less desirable, considering that it just recapitulates some of the CD symptoms, and above all, it does not represent changes deriving from a long-term disease.

By adopting this model, the PTG administration for ten days to our rats previously sensitized by IFN-γ led to an evident modification of the intestinal mucosa with a significant reduction of the length of villi.

The histological alterations caused by PTG treatment were accompanied by an increase, although not significant, in the polyamine content as well as in the ODC activity and expression in the jejunal tissue samples. This finding agrees with our data from previous work on Caco-2 cells [18] and confirm the ability of gliadin to induce the expression and activity of arginase. This enzyme causes the transformation of arginine into ornithine and urea, and, consequently, induces the ODC activity leading to the production of polyamines [34]. Nonetheless, the protective role of polyamines on the damage of the intestinal epithelia caused by gliadin administration is still controversial, and different mechanisms of action have been hypothesized, including their effect on the functions of intestinal brush border or intracellular membranes involved in the handling of gliadin [35]. Moreover, the increase in cell proliferation during the repair phase of mucosal damage could cause an increase in the total polyamines. These polycations are also considered reliable markers of hyper-proliferation and their enhancement following the toxic gliadin effect, could be similar to that observed during other inflammatory processes of the intestine leading to a derangement of the intestinal mucosa [36].

PTG treatment also caused a significant reduction in the mRNA of all the tested TJs and AJs in the jejunal tissue samples followed by a concomitant, although not significant decrease in their protein levels. It is well known that the intercellular junctions between intestinal epithelial cells are abnormal in CD patients [37]. Celiac individuals have fewer TJ protein strands and less of the TJ-associated protein ZO-1. Also, the AJ proteins required for TJ formation result in be reduced in the duodenal epithelium of children with CD.

Apart from the proposed ongoing new therapeutic solutions for CD (including genetically modified gluten, zonulin inhibitors, therapeutic vaccines, and tissue transglutaminase inhibitors), probiotics appear to be a practical, integrative therapeutic approach to CD management along with a gluten-free diet [38]. According to the FAO/WHO, a probiotic is defined as a “live microorganism, which when administered in adequate amounts confers a health benefit on the host” [39]. Their beneficial effects on the gut health have been widely investigated and concern the production of inhibitory substances against pathogens, the blockage of adhesion sites, the competition for nutrients, the degradation of toxin receptors, and the regulation of immunity [40]. Nevertheless, the benefits for CD patients following probiotic administration and the molecular mechanisms supporting its action still need to be characterized. A list of mechanisms of actions have been hypothesized that includes the capacity of some probiotic strain to hydrolyze gliadin [41], or split it into smaller nontoxic peptides by particular peptidases [42]; additionally, probiotics might inhibit the entrance of gliadin peptides directly into the epithelial cells [43].

In our set of experiments, the exposure to L.GG did not exert any significant effect on the rat intestinal mucosa which appeared very similar to that of controls. On the contrary, when rats were sensitized with IFN-γ and co-administered with PTG and L.GG for ten days, a restoring of the damaged mucosa was evident with a significant increase of the length of villi compared to PTG treated rats. Of note, the repairing of the injured mucosa was not so efficient in the group of rats that were sensitized with IFN-γ, pre-treated with PTG for ten days and then administered with L.GG for further ten days. These animals showed still an altered mucosa with the presence of some inflammatory cells between the villi and on the surface of the mucosa. Although thickness of the mucosa resulted in be not increased, some areas showed a cellular double layer probably due to proliferative abnormalities. This finding may be related to the need to increase the jejunal absorptive surface area [44], supporting once more the concept of a tissue reaction to the detrimental effect induced by PTG.

As for the polyamine metabolism, L.GG administration for ten days to rats sensitized with IFN-γ led to a significant reduction in the ODC activity and expression in the jejunal tissue samples. Besides, the co-administration of L.GG and PTG to rats opposed the effects of PTG on the polyamine metabolism, leading to the significant decrease of the total polyamine levels. These findings agree with our previous data about the relationship between polyamine metabolism and probiotics during carcinogenesis and tumor growth demonstrating their antiproliferative action [45].

The intercellular junction protein expression was investigated, and the L.GG exposure to rats did not cause any significant effect, except for a significant increase in the ß-catenin protein levels. Besides, when the probiotic strain was co-administered with PTG, a significant induction in the mRNA levels of all the tested genes was observed. Besides a concomitant increase in the ß-catenin and E-cadherin proteins was observed.

Experimental evidence that many commensal bacteria can modulate epithelial permeability derived from studies with probiotics in various disease models. However, the administration of different probiotic strains seems not to modify the intestinal epithelial permeability in healthy control animals [10], and this finding suggests that the presence of probiotics is essential for the prevention of intestinal barrier dysfunction only upon injury. Our present results seem in agreement with this hypothesis and suggest that L.GG may have a protective effect when co-administered with the toxic agent but also a therapeutic action once PTG has caused the damage to the intestinal mucosa.

Previous in vitro studies by our group have already reported the potential of L.GG in repairing the cell barrier function by modulating the trans epithelial resistance (TER), the release of zonulin and the intercellular junction protein expression [17,18]. Improvements in barrier integrity are associated with changes in TJ structure via changes in TJ protein expression and distribution [46,47]. Existing mechanistic studies have focused on the ability of probiotics to prevent alterations to a few TJ bridging proteins in disease models, e.g., the effects of VSL#3 on dextran sodium sulphate-induced colitis in mice [48] and *L. plantarum* CGMCC 1258 on *Enteroinvasive E. coli* EIEC—ATCC 43893 (serotype O124:NM)-induced barrier disruption in vitro [49]. Overall, it is accepted that a class of TJ-strengthening probiotics including L.GG could up-regulate the expression of TJ proteins (notably Occludin and ZO-1) or down-regulate the expression of the pore-forming protein Claudin-2, thus supporting that probiotics directly regulate TJ barrier function at the gene expression level [50,51]. Furthermore, probiotics have been reported to modulate various protein kinase signaling pathways that can enhance phosphorylation of TJ proteins, including Rho family, GTPases, PKC and MAPK. For example, the ability of the probiotics *Streptococcus thermophilus* and *Lactobacillus acidophilus* to preserve the phosphorylation of occludin in EIEC-infected cells could be reduced by treating the cells with Rho kinases (ROCK) inhibitors [52], suggesting these bacteria employ Rho family GTPases to protect against EIEC-induced TJ disruption. 

Interestingly, the positive role of L.GG was further confirmed when this probiotic was administered to rats after the establishment of the damage induced by ten days exposure to PTG. Noteworthy, the significant induction in the mRNA levels of all the tested intercellular junction proteins was even more evident and all the evaluated protein levels significantly increased. This stimulation of the expression of the intercellular junction proteins might be explained by a fast response of the epithelial cells to the damage induced by PTG treatment before probiotic administration. In fact, the PTG treatment, causing defects in TJ and AJ proteins, might lead to changes in epithelial morphology and integrity and potentially to faster trafficking of inflammatory cells through the epithelium, as demonstrated by our histological evaluation [53].

In literature, published data are mainly based on a prior or concomitant probiotic administration with different harmful agents tested. Infection with EIEC caused a significant reduction in serine phosphorylation of occludin and tyrosine phosphorylation of ZO-1, without a substantial change in the abundance of these proteins [50]. These harmful effects were reduced by live probiotic pre-treatment or simultaneous treatment, in the latter case only at high concentrations. 

## 5. Conclusions

In conclusion, this probiotic strain acts by preventing the reduction of the expression of the intercellular junction proteins. According to available data in the literature and based on our present results, we could suppose that under normal conditions, L.GG cannot alter the intercellular junction proteins. This probiotic strain could instead bring an increase in the gene expression of TJs and AJs once the damage has been induced. The event could happen not only if L.GG is co-administered with the PTG but also, and more dramatically if the probiotic is added some days after the treatment with PTG. Thus, we can hypothesize that L.GG may decrease intestinal permeability only when some damaging agent, such as PTG, is modifying or has already modified the intestinal barrier function. However, the limitation of the animal model used to reproduce the fundamental features of CD adequately makes necessary to consider the reported results with caution, and, undoubtedly, studies on humans will be required to prove the beneficial effects of this bacterium on the disease. However, our present data let us hypothesize the use of L.GG in the therapeutic management of the gluten-related disorders in humans.

## Figures and Tables

**Table 1:** Morphometric evaluation of jejunal tissue sections from control and treated rats.

	Villi	
	Length (µm)	Width (µm)
Ctrl	259.26 ± 1.98	50.59 ± 0.44
PTG	144.41 ± 2.84 **	45.02 ± 0.40
L.GG	260.11 ± 0.91	53.91 ± 0.26
Co-administered	221.64 ± 1.71 ^§^	54.92 ± 0.54
Pre-treated	159.14 ± 1.98 **	50.22 ± 0.25

Ctrl (without treatment); PTG (sensitized with 1000 U IFN-γ and treated with PTG 50 µg/day for 10 days); L.GG (treated with L.GG 1 × 10^9^ CFU for 10 days); Co-administered (sensitized with 1000 U IFN-γ and co-administered with PTG and L.GG for 10 days); Pre-treated (sensitized with 1000 U IFN-γ, pre-treated with PTG for 10 days, and then administered with L.GG for further 10 days). All data are expressed as mean ± SEM of 20 independent microscopic fields of each animal (n = 3) and were analysed by Kruskal-Wallis analysis of variance and Dunn’s Multiple Comparison Test. (**) *p* < 0.01 compared to control rats. (^§^) *p* < 0.05 compared to PTG treated rats.

**Table 2:** Polyamine profile in jejunal tissue samples from control and treated rats.

	Putrescine	Spermidine	Spermine	Total Polyamines
Ctrl	2.32 ± 0.3	12.54 ± 1.0	7.72 ± 0.5	22.59 ± 1.8
PTG	1.89 ± 0.1	14.88 ± 0.5	8.42 ± 0.4	25.20 ± 0.7
L.GG	1.92 ± 0.2	11.05 ± 0.3	7.82 ± 0.1	20.79 ± 0.5
Co-administered	1.94 ± 0.2	10.68 ± 0.6 **^§^**	8.53 ± 0.3	21.16 ± 1.1 **^§^**
Pre-treated	1.86 ± 0.1	11.58 ± 0.7	7.57 ± 0.3	21.01 ± 0.6 **^§^**

Ctrl (without treatment); PTG (sensitized with 1000 U IFN-γ and treated with PTG 50 µg/day for 10 days); L.GG (treated with L.GG 1 × 10^9^ CFU for 10 days); Co-administered (sensitized with 1000 U IFN-γ and co-administered with PTG and L.GG for 10 days); Pre-treated (sensitized with 1000 U IFN-γ, pre-treated with PTG for 10 days, and then administered with L.GG for further 10 days). All data represent the results of three different experiments (mean ± SEM) and were analysed by Kruskal-Wallis analysis of variance and Dunn’s Multiple Comparison Test. Polyamines are expressed as nmol/mg protein. (^§^) *p* < 0.05 compared to PTG treated rats.
